# Case Report: A Calculus-Free Ureteral Stent Forgotten for 29 Years

**DOI:** 10.3389/fsurg.2022.878660

**Published:** 2022-04-29

**Authors:** Cheng Tang, Genyi Qu, Guang Yang, Gang Wang, Yong Xu

**Affiliations:** ^1^Department of Urology, The Affiliated Zhuzhou Hospital of Xiangya Medical College, Central South University, Zhuzhou, China; ^2^Department of Rheumatology and Immunology, The Affiliated Zhuzhou Hospital of Xiangya Medical College, Central South University, Zhuzhou, China

**Keywords:** urology, case report, ureteral stent, endoscopy, forgotten, no calculus

## Abstract

Ureteral stents are widely used. If ureteral stents remain in place for extended periods, the probability of migration and stone formation increases substantially. However, a 29-year-old ureteral stent that was placed and did not develop calculus is rare. We reported a 45-year-old man admitted with pain in the left side of his waist and abdomen for more than 10 years. He underwent a ureterotomy 29 years prior to admission for left ureteral calculi, and a ureteral stent was placed postoperatively to prevent ureteral strictures. The ureteral stent was not removed in the hospital due to poor compliance on the part of the patient. This left ureteral stent was not visible on computed tomography (CT) and plain films. On ureteroscopy or flexible ureteroscopy, no new calculus was found in the left ureter and kidney. No calculus was found in the stent that is usually easily removed with calculus-removing forceps. This phenomenon is rare, and it highlights the importance of follow-up.

## Introduction

Ureteral stents are widely used to treat renal and ureteral obstructive diseases to maintain smooth urine drainage and protect renal function (1). Ureteral stents are used in renal and ureteral calculi surgery to promote the expulsion of calculi fragments and prevent ureteral strictures. Severe complications, such as stent encrustation (2–6), stent migration (7), fragmentation (4), stone formation (2, 5, 8), hydronephrosis (9), and urosepsis (10), may appear when the stent has been left *in situ* for extended periods. If a ureteral stent remains for more than 6 months, the probability of migration and calculus formation of the stent increases substantially (11). Although composite materials such as a mixture of polyethylene, polyurethane, and silicone have been studied in ureteral stents (12, 13), the incidence of stent encrustation has not significantly reduced. We present the case of a ureteral stent forgotten for 29 years that did not form a stone.

## Case Report

A 45-year-old man was admitted to the hospital with intermittent pain in the left side of his waist and abdomen for more than 10 years. He occasionally complained of hematuria that resolved without intervention. Left upper ureteral calculi were removed by open surgery 29 years before admission. He had a history of hypertension for 3 years, and his blood pressure was reasonably well-controlled. The patient ignored the condition because of the limited level of local medical services and his pain tolerance. A recent B-ultrasound revealed left hydronephrosis consistent with left urinary tract obstruction. The patient was admitted to our hospital.

**Figure 1:**
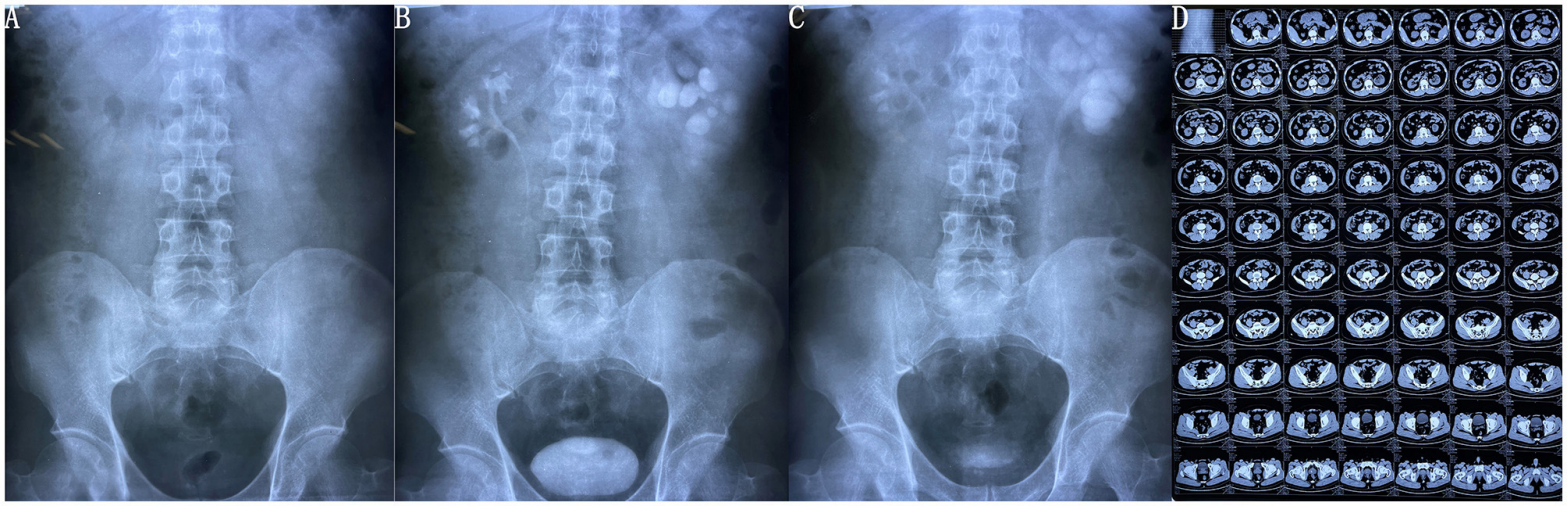
Preoperative kidney ureter bladder (KUB) film, intravenous urography (IVU), and abdominal CT. **(A)** There was no evident stent image on preoperative KUB; **(B,C)** show the preoperative IVU revealing left hydronephrosis, delayed left renal excretion, dilation of the left ureter, and no evident obstruction in the left ureter; **(D)** absence of evident stent on preoperative abdominal CT.

Examination of the left kidney area revealed mild percussive pain. Urinary system B-ultrasound revealed a few hypoechoic masses in the lower part of the left ureter, kidney calculus, left hydronephrosis, and the presence of some implants in the left ureter. On further questioning, the patient recalled that a stent had been placed in the left ureter following an open procedure for ureteral calculus; however, he neglected to return to the hospital to have the stent removed. His urea nitrogen was 8.02 mmol/L, and creatinine was 135.8 μmol/L. Triglycerides were 4.95 mmol/L. Microscopic hematuria was seen on routine urine examination (25 cells/μL). Urine white blood cells and nitrite were negative, and urine pH was 5.6. There were no abnormal indexes in other routine blood tests, liver function, electrolytes, or urine culture. It was challenging to distinguish the ureteral stent both on kidney ureter bladder (KUB) film (Figure 1A) and non-contrast computed tomography (CT) (Figure 1D). Preoperative intravenous urography (IVU) revealed left hydronephrosis, delayed left renal excretion, dilation of the left ureter, and no evident obstruction in the left ureter (Figures 1B,C). Ureteroscopy was planned to perform left ureteral exploration.

**Figure 2:**
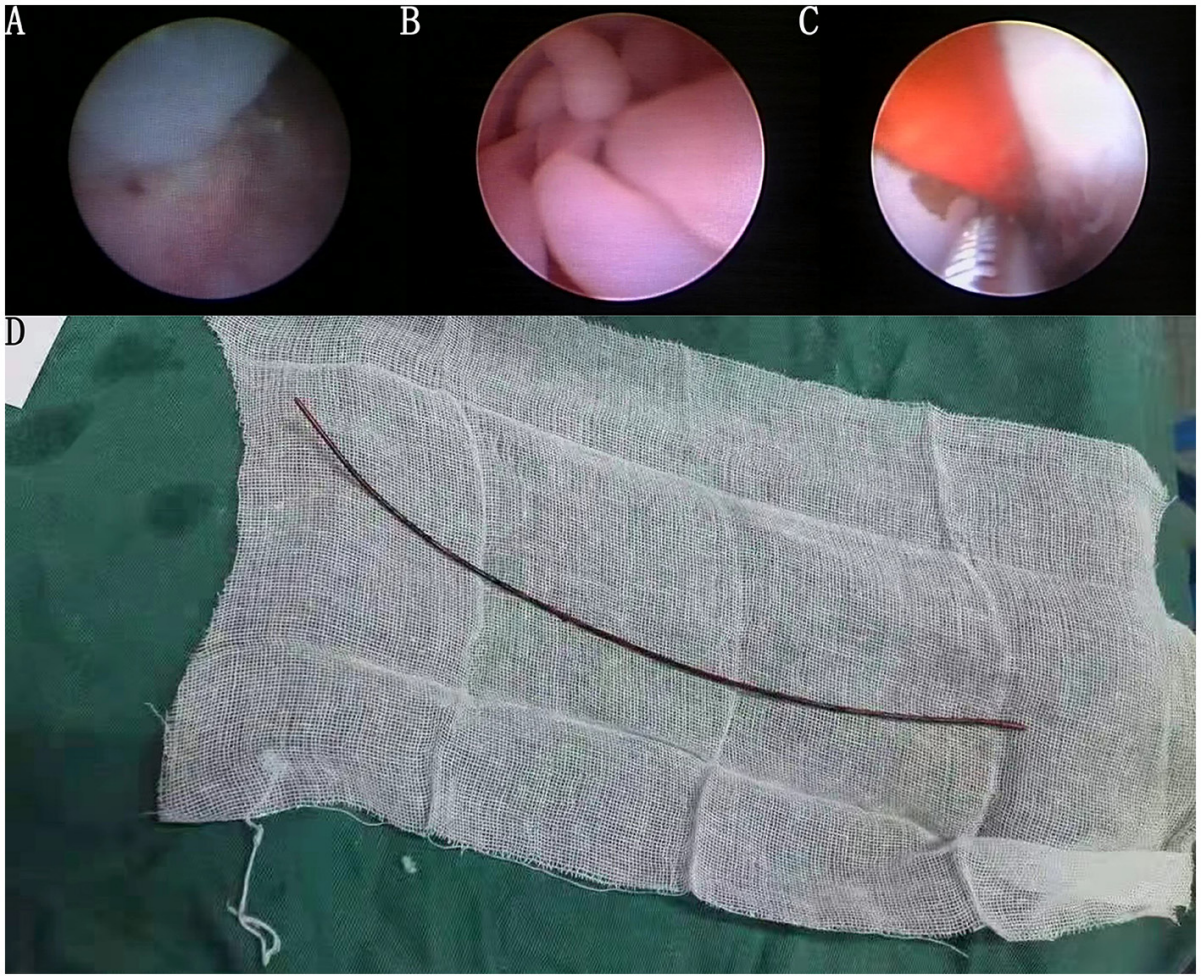
Many polyps in the left ureter and the appearance of the ureteral stent. **(A)** Many white polyps at the entrance of the left ureter are seen via the ureteroscope. **(B)** Many white polyps in the middle of the left ureter are seen via the ureteroscope. **(C)** Orange ureteral stent observed during ureteroscopy. **(D)** Unbroken ureteral stent removed successfully.

**Figure 3:**
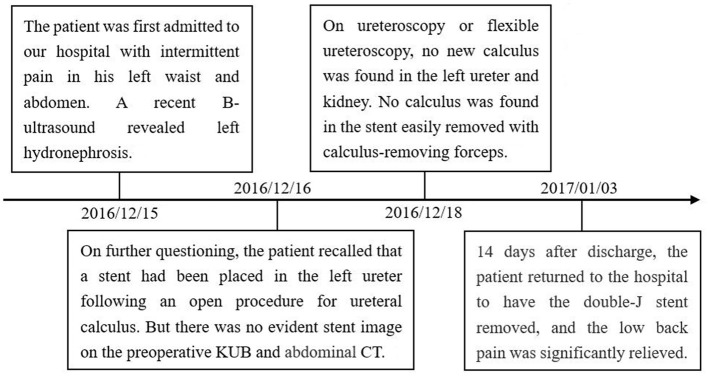
Timeline of symptoms, treatment, and prognosis in our case.

We performed preoperative preparations and operated with the patient in the lithotomy position under intravenous and inhaled general anesthesia. A ureteroscope (Wolf F8.0/9.8) was used to enter the bladder through the urethra, and many polyps near the left ureteral orifice were seen (Figure 2A). Using a zebra guidewire, the ureteroscope was advanced into the left ureter, and several polyps in the ureter were seen (Figure 2B). After withdrawing the ureteroscope, the guidewire remained in place. A flexible ureteral sheath was delivered to the upper part of the left ureter using the guidewire, and a flexible ureteroscope (Olympus 8F/2.65 mm) was inserted. An orange ureteral stent was discovered in the left ureter, and there was no evident calculus around the stent. Calculus removal forceps were used to remove the stent (Figure 2C). The stent had a diameter of about 2 mm and a length of 23 cm (Figure 2D). A novel 6-F double-J stent was placed in the left ureter. The procedure required 30 min and ended uneventfully. After 14 days, the patient returned to the hospital to have the double-J stent removed, and the low back pain was significantly relieved. The patient thought that it is necessary to seek medical attention in a timely manner. The timeline of the patient's symptoms, treatment, and prognosis in our case is shown in Figure 3.

## Discussion

Ureteral stents are commonly used in urological procedures to maintain smooth drainage. In 1976, Gibbons et al. explained the use of indwelling ureteral stents (14). Stents are usually placed for 2 to 12 weeks (11). Newer ureteral stents can remain in place for more extended periods (6 months to 1 year) (15). However, the stent itself is a foreign object that causes a series of rejection reactions. Short-term complications of the ureteral stent include hematuria, low back pain, bladder irritation, and stent displacement (16, 17). The attending physician should advise the patient to return to have the stent removed. Short-term complications are often significantly relieved after removing the ureteral stent. Nevertheless, some patients still have poor compliance and forget to return for removal (sometimes for more than 10 years). In the long term, severe complications might ensue, including stent encrustation (2–6), novel calculus formation (2, 3, 5, 7), stent migration (18), stent fragmentation (6, 19, 20), hydronephrosis (2, 9, 21), or urosepsis (10). A literature search revealed that stent encrustation and urinary stone formation are the most common long-term complications. Kawahara et al. found the rate of ureteral stent encrustation was 26.8% within 6 weeks, 56.9% between 6 and 12 weeks, and 75.9% over 12 weeks (22). Surgery is often required for treatment, and combinations of surgical modalities are performed if necessary. Some case reports are shown in Table 1.

**Table 1:** Available case reports found on literature search.

**Author**	**Age and sex**	**Placement time**	**Complications**	**Interventions**
Pühse et al. (21)	A 44-year-old man	17 years	Left hydronephrosis andmassive stones in the left kidney and bladder	Heminephroureterectomy, right-sided pyelotomy, and midline vesicostomy
Nikkhou et al. (4)	A 40-year-old man	5 years	Encrusted and fractured ureteral stent, left kidney, and ureteral stones	Left ureterorenoscopy with laser lithotripsy and basket extraction of stent fragments and stones.
Singh et al. (5)	A 27-year-old man	9 years	Encrusted and fractured ureteral stent and complex panurinary stone	Cystoscopy lithotripsy and percutaneous nephrolithotripsy
Kawahara et al. (18)	A 69-year-old woman	3 years	Ureteral stent migration	Ureteroscopic stent removal surgery
Barreiro et al. (10)	A 73-year-old woman	3 years	Encrusted and fractured ureteral stent, massive stones in left kidney and bladder, urinary incontinence, and urosepsis	Open left nephrectomy and cystolithotomy
Bidnur et al. (3)	A 36-year-old woman	12.5 years	Encrusted ureteral stent and massive stones in left kidney and bladder	Percutaneous nephrolithotomy cystolitholapexy, and ureteroscopy
Tao et al. (20)	A 13-year-old man	4 years	Encrusted and fractured ureteral stent and massive stones in the bladder	Percutaneous nephrolithotomy combined with suprapubic cystolithotomy
Mahmood et al. (6)	A 28-year-old man	15 years	Encrusted and fractured ureteral stent and massive stones in right kidney and bladder	Percutaneous nephrolithotomy and ureteroscopy
Nesbitt et al. (23)	A 26-year-old man	26 years	Encrusted ureteral stent	Ureteroscopy with stone extractor basket
Gill et al. (9)	A 29-year-old woman	1 year	Left hydronephrosis and bladder stones	Cystoscopy extensive lithotripsy with holmium laser
Zhang et al. (2)	A 45-year-old woman	6 years	Encrusted ureteral stent, massive stones in the bladder, and right hydronephrosis	Pneumatic ballistic lithotripsy
Kim et al. (8)	A 43-year-old woman	25 years	Ureteral stent with extensive calcifications and severe left hydronephrosis	Laparoscopic nephroureterectomy
Al-Hajjaj et al. (19)	A 52-year-old man	2 years	Fractured ureteral stent and bladder stones	Cystoscopy and ballistic lithotrites
Aboutaleb et al. (7)	A 49-year-old man	10 years	Multiple ureteral stones and bladder Calculus and encrusted and migrated ureteral stent	Cystolithotripsy, ureteroscopic laser lithotripsy, and stent removal

Kim et al. (8) reported a female patient whose ureteral stent had been forgotten for 25 years and developed a sizable ureteral stent stone; a laparoscopic nephroureterectomy was performed to remove the stent. Nesbitt et al. reported a ureteral stent forgotten for 26 years; however, the stent was mildly encrusted (23). Another case of ureteral stent migration was reported by Kawahara et al.; this 3-year-old stent was easily removed under fluoroscopic observation (18). Our case was of a ureteral stent *in situ* for 29 years; to the best of our knowledge, there are no reports of ureteral stents left for this long. A remarkable finding was the absence of stent encrustation or novel calculus formation. This finding differs from the majority of forgotten cases of ureteral stents. Although there were polyps around the stent, the stent was removed entirely.

Zhang et al. reported a forgotten case of a ureteral stent for 5 years (2). A large bladder calculus had formed at the end of the double-J stent in the bladder. There was no evident stone on the remaining ureteral stent. Although the time that the ureteral stent remained was shorter than the present case, the author mentioned that renal insufficiency of the affected side led to decreased urine secretion, which may explain why the ureteral stent did not show evident calculi formation. This finding was similar to our study in which the patient had moderate left hydronephrosis (2).

In infected and sterile urine, the deposition of encrusted material on retained ureteral stents can occur. The rate of encrustation depends on urine composition, infection status, and metabolic or congenital abnormalities (3, 18). Another possibility is the nature of the material of this ureteral stent. Because we did not contact the surgeon who performed the initial procedure, we cannot provide specific information regarding this ureteral stent. We speculate that this stent may be the pusher for a traditional double-J stent with a relatively smooth surface that is not conducive to calculus formation. No evident trace of this ureteral stent was found on non-contrast CT or KUB; this finding also differed from other case reports. The lack of imaging evidence may be explained by the similar density of the ureteral stent and the surrounding soft tissue. We will further analyze the specific composition of this stent material.

Patient education is critical to prevent forgotten ureteral stents. However, some ureteral stents cannot be removed quickly (24). It is essential to emphasize the importance of follow-up. A ureteral stent register system might help avoid forgotten ureteral stents (25).

We reported the most prolonged interval of a forgotten ureteral stent. The most remarkable finding was the absence of new calculus formation on the stent or the affected kidney. An analysis of the specific components of this stent might help to improve the materials used in such stents. It cannot emphasize more the importance of follow-up in patients.

## Data Availability Statement

The original contributions presented in the study are included in the article/supplementary material, further inquiries can be directed to the corresponding author/s.

## Ethics Statement

Written informed consent was obtained from the individual(s) for the publication of any potentially identifiable images or data included in this article.

## Author Contributions

CT collected the data and wrote the main manuscript text. YX edited the manuscript and performed the surgery. GQ collected the data and took a video of the operation. GY and GW collected the data and edited the manuscript. All authors substantially contributed to the approval of the final version of the manuscript.

## Conflict of Interest

The authors declare that the research was conducted in the absence of any commercial or financial relationships that could be construed as a potential conflict of interest.

## Publisher's Note

All claims expressed in this article are solely those of the authors and do not necessarily represent those of their affiliated organizations, or those of the publisher, the editors and the reviewers. Any product that may be evaluated in this article, or claim that may be made by its manufacturer, is not guaranteed or endorsed by the publisher.
